# Socioeconomic correlates of urban mobility trends in two Australian cities during transitional periods of the COVID-19 pandemic

**DOI:** 10.1098/rsos.241463

**Published:** 2025-01-15

**Authors:** Pratyush Kollepara, Subhrasankha Dey, Martin Tomko, Erika Martino, Rebecca Bentley, Michele Tizzoni, Nicholas Geard, Cameron Zachreson

**Affiliations:** ^1^ School of Computing and Information Systems, The University of Melbourne, Parkville, Victoria, Australia; ^2^ Department of Mathematical and Physical Sciences, La Trobe University, Melbourne, Victoria, Australia; ^3^ Department of Built Environment, Aalto University, Espoo, Finland; ^4^ Department of Infrastructure Engineering, The University of Melbourne, Parkville, Victoria, Australia; ^5^ Centre for Health Policy, Melbourne School of Population and Global Health, The University of Melbourne, Parkville, Victoria, Australia; ^6^ Centre of Research Excellence in Health Housing, Melbourne School of Population and Global Health, The University of Melbourne, Parkville, Victoria, Australia; ^7^ Department of Sociology and Social Research, University of Trento, Trento, Italy

**Keywords:** COVID-19, nonpharmaceutical interventions, mobility data, behaviour change, socioeconomic

## Abstract

During the COVID-19 pandemic, both government-mandated lockdowns and discretionary changes in behaviour combined to produce dramatic and abrupt changes to human mobility patterns. To understand the socioeconomic determinants of intervention compliance and discretionary behavioural responses to epidemic threats, we investigate whether changes in human mobility showed a systematic variation by socioeconomic status during two distinct periods of the COVID-19 pandemic in Australia. We analyse mobility data from two major urban centres and compare the trends during mandated stay-at-home policies and after the full relaxation of nonpharmaceutical interventions, which coincided with a large surge of COVID-19 cases. We analyse data aggregated from de-identified global positioning system trajectories, collated from providers of mobile phone applications and aggregated to small spatial regions. Our results demonstrate systematic decreases in mobility relative to the pre-pandemic baseline with the index of education and occupation, for both pandemic periods. On the other hand, the index of economic resources was not correlated with mobility changes. This result contrasts with observations from other national contexts, where reductions in mobility typically increased strongly with indicators of wealth. Our analysis suggests that economic support policies in place during the initial period of stay-at-home orders in Australia facilitated broad reductions in mobility across the economic spectrum.

## 1. Introduction

Equitable public health responses to pandemics such as COVID-19 require an understanding of the socioeconomic correlates of behaviour associated with intervention policies and epidemic dynamics [1]. Public health interventions put in place during the COVID-19 pandemic affected human mobility across all spatial scales, from the effects of international travel restrictions to the micro-distancing policies put in place to decrease crowd density in public spaces [2]. Owing to the widespread use of mobile devices, changes in mobility that occurred during COVID-19 have been the subject of unprecedented levels of analysis and have proved useful for understanding the many factors influencing behaviour change during the pandemic [3–11]. Furthermore, these behaviour changes have been shown to be associated with changes in transmission risk [6,7,12,13].

Research on the relationship between changes in mobility during COVID-19 lockdown periods as a function of socioeconomic status (SES) has been conducted in many countries, using mobility data or through self-reported activity surveys. Some studies use a single measure of SES while others consider various components of SES such as education, occupation and income. Studies that use a single aggregate measure of SES have reported that mobility or behaviour change is the least pronounced for those with low SES [14–16]. Studies that consider the components of SES indices have found differing trends of mobility with the SES components, such as a decrease in mobility with income [17–19], decrease in mobility with education but no variation with income [20,21], decrease in mobility with income but no variation with education level [22] and decrease in mobility with both wealth and occupational status [23]. While such studies demonstrate that socioeconomic conditions are important considerations for understanding and predicting behavioural responses to interventions, the lack of consensus illustrates the contextual complexity of these questions. Our study aims to analyse complex drivers of movement behaviour by comparing periods exhibiting contrasting behavioural stimuli.

In this study, we investigate mobility changes within Australian urban centres during two phases of the pandemic. We aim to understand the differences between the behavioural response to mandated stay-at-home policies, implemented during the early phases (April 2020), and the response to the full relaxation of those policies which occurred almost two years later (January 2022). Our study uses de-identified global positioning system (GPS) data, collated from providers of mobile phone applications and aggregated to small spatial regions by the Australian data analytics company *Pathzz*. By assessing changes to mobility levels from each region as a function of socioeconomic characteristics (assessed by the Australian Census), we identify socioeconomic drivers of mobility behaviour change during two distinct periods of the COVID-19 pandemic in Australia.

**Figure 1.**
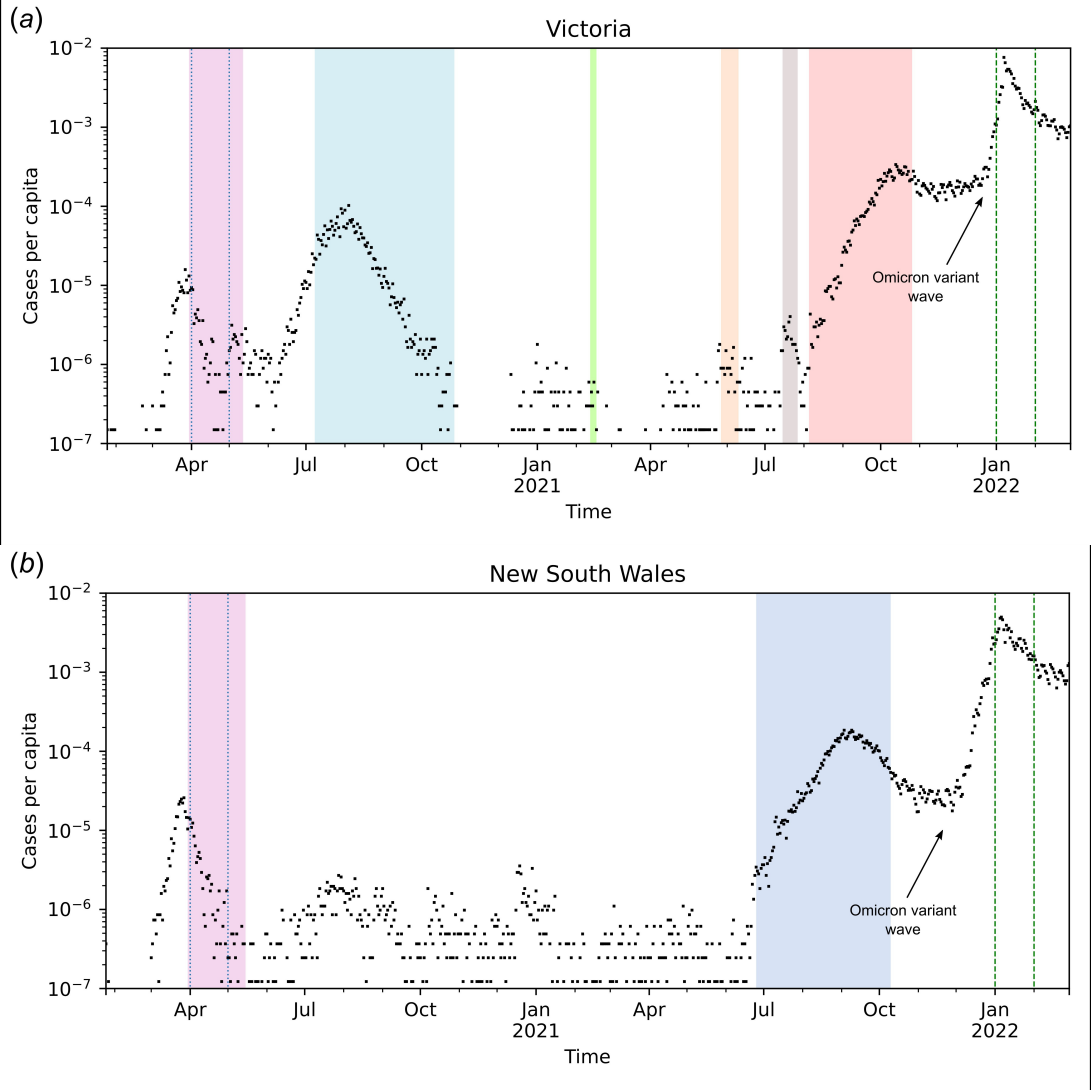
A timeline of COVID-19 cases and stay-at-home policies in the states of Victoria (*a*) and New South Wales (*b*) in Australia [24,25]. The plot shows daily new cases per capita. The coloured patches show the various intervention periods. The two pairs of vertical lines show the periods for which we analysed mobility trends: April 2020 and January 2022. The timelines of stay-at-home policies were obtained from an Australian parliamentary report, news articles and media releases from state governments [26–32].

Our investigation focuses on the two largest Australian cities (Sydney and Melbourne), and we examine two scenarios: the initial period of stay-at-home orders, and the re-opening phase that coincided with a wave of infections caused by the Omicron variant. Mandated stay-at-home policies were introduced at the end of March 2020 (figure 1). During this period, disease prevalence was low, risk perception was almost universally high and stay-at-home orders were supported by the Australian Government both through financial support packages and through large financial penalties introduced to enforce the measures [26,33]. In the second scenario (re-opening), stay-at-home orders were universally relaxed, along with all other travel restriction policies introduced throughout the pandemic. Though stay-at-home orders were not in effect, some prevention policies were in place (scanning QR codes for contact tracing, mask wearing in certain spaces, etc.; [26–28]). During this period, risk perception remained high for large sections of the population. Policy relaxation, combined with the emergence of the newly dominant Omicron variant, produced a surge in confirmed COVID-19 cases in January of 2022. This surge in cases resulted in what was referred to as ‘shadow lockdown’, i.e. substantial discretionary reductions in movement and social activity resulting from the perceived risk of infection [34,35]. (Note: the term ‘shadow lockdown’ should not be confused with the similar term ‘shadow pandemic’, which is used in other work regarding domestic violence during COVID-19 [36]).

**Figure 2.**
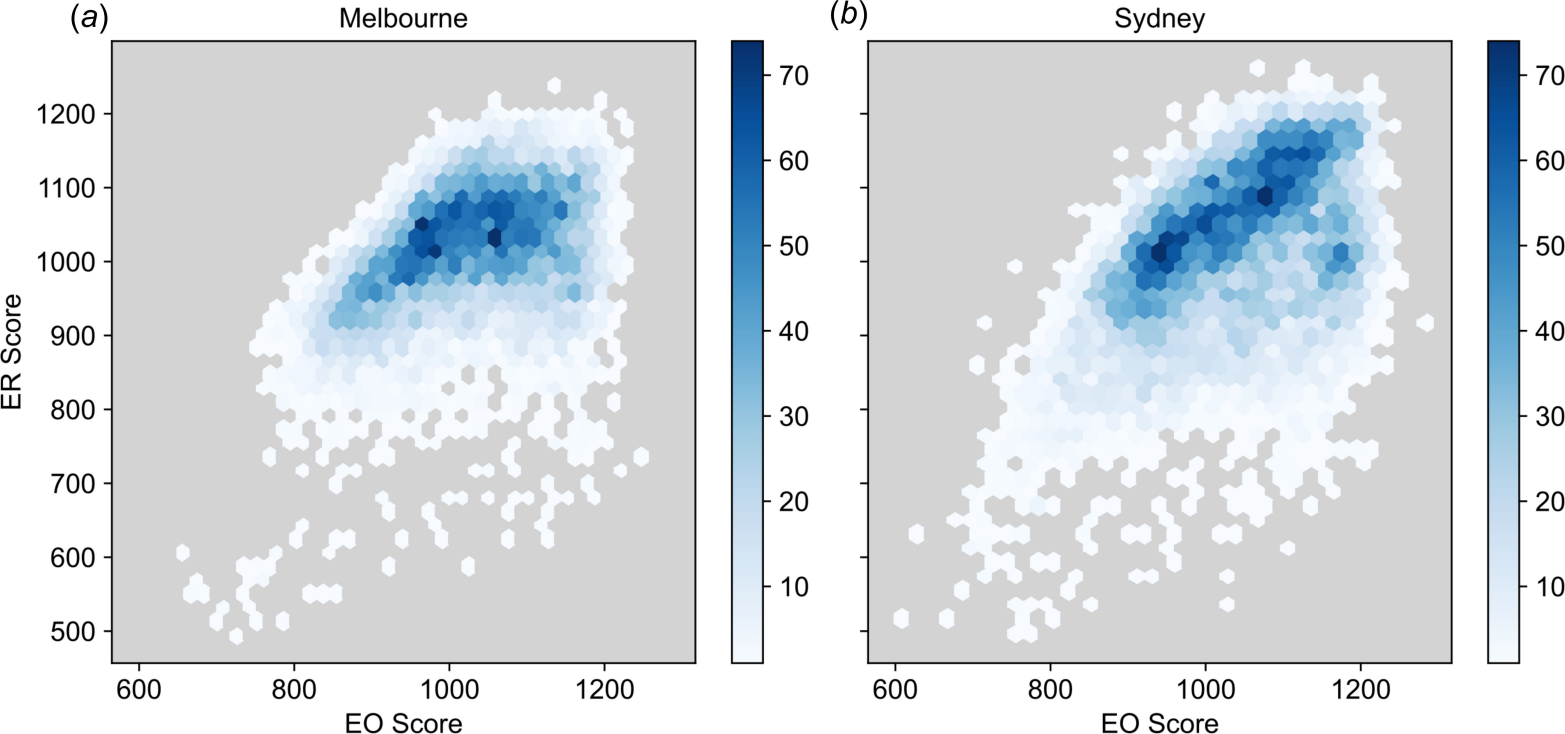
Joint distribution of index of EO and index of ER scores in (*a*) Melbourne and (*b*) Sydney. The colour bar shows the number of statistical areas in the hexagonal bins of the histogram.

To characterize socioeconomic variation in Sydney and Melbourne, we use two aggregate indices of SES, which characterize broadly distinct categories of population features. Specifically, one of the indices exclusively quantifies economic resources (ER) while the other combines education and occupational (EO) factors. While correlated (figure 2), these indices are formed from separate sets of component variables measured by the Australian Census [37]. To study if and how changes to mobility behaviour varied along socioeconomic lines, we examine median trends in mobility for population strata defined by these socioeconomic indices. While this forms the basis for the results, we test the robustness of this qualitative analysis with three statistical models which include possible confounding variables and spatial effects.

Our findings demonstrate that, for both periods, economic resources were not associated with differences in mobility behaviour. On the other hand, EO factors were associated with reduced mobility during both the initial period of stay-at-home orders, and the re-opening phase (coinciding with the Omicron wave). Notably, these differences in mobility behaviour with socioeconomic indicators occur despite the fact that EO and ER indices are correlated, requiring an analysis of mobility across their joint distribution. This joint analysis demonstrates that the effects of ER (increasing mobility) competed with those of EO (decreasing mobility), producing a trend in which regions with high EO index, but low ER index exhibited lower levels of mobility compared to those with low EO and high ER.

## 2. Methods

### 2.1. Scenario descriptions

#### 2.1.1. Pandemic conditions in April 2020 and January 2022

Before the introduction of stay-at-home orders, daily counts of confirmed COVID-19 infection increased through most of March 2020, with the trend reversing by the end of March in both Victoria and New South Wales (NSW) and more than 500 and 1000 daily cases reported at peak levels in the two states. This reversal coincided with stay-at-home orders and international travel restrictions. By the end of March 2020, a total of 2153 cases had been detected in NSW and 956 cases had been detected in Victoria. During April 2020, the first period we analysed in our study, case counts broadly declined in both states. By the end of April, 3018 cases had been detected in NSW and 1365 in Victoria. During the second period of interest, January 2022, daily case counts were in the order of 10^5^ in both states, owing to the recent introduction of the Omicron variant and relaxation of restrictions [38].

#### 2.1.2. Policy conditions in April 2020 and January 2022

In Victoria, activity restrictions to combat transmission of COVID-19 had increased in intensity over time to include: banning non-essential gatherings of size more than 500 persons, banning indoor gatherings of more than 100 persons, restrictions on visits to aged care homes, and closure of non-essential services. On 31 March 2020, ‘stage three’ restrictions were announced. Only four reasons were prescribed as a justification for an individual to leave their home: to acquire food and supplies, to access healthcare, to exercise, and for the purpose of work/education. Gatherings of more than two persons were not allowed unless the individuals belonged to the same household and for work/education. Playgrounds, skateparks and outdoor gyms were closed. On-the-spot fines of AUD$1652 (worth minimum wage work of 71 h) for individuals and AUD$9913 for businesses were enforced if any of these restrictions were breached. All schools transitioned to remote and flexible teaching [26].

In NSW, activity restrictions included: banning non-essential indoor gatherings of more than 100 persons, and outdoor gatherings of more than 500 persons, restrictions on visits to aged care homes, enforced social distancing of 1.5 m and closure of non-essential businesses and activities. On 31 March 2020, NSW issued a set of valid reasons for leaving one’s home, as did Victoria [26,39]. Relief measures during this time included a national moratorium on evictions and monetary payments to more than 5 million people, among other measures and support for housing [26,33].

Between April 2020 and December 2021, Victoria had gone through five subsequent lockdown periods of varying intensity and duration, while NSW had one subsequent lockdown. By January 2022, major restrictions on movement and gatherings were removed across the country (conditional on vaccination), though density limits and mask mandates remained [29–32].

#### 2.1.3. Baseline periods

The baseline periods used for comparing the mobility during the periods of interest were: 15 September–15 October 2019 (for comparison with April 2020, the lockdown period) and January 2020 (for comparison with January 2022, the Omicron wave period) (table 1). Annually matched mobility data from April 2019 was not available for comparison with April 2020, so we selected the period between 15 September and 15 October because it has a similar number of school holiday dates which we expect to influence mobility patterns. In January 2020, Australian states detected their first COVID-19 cases but interventions were not in place other than screening procedures at international airports [26].

**Table 1.** Periods of interest and the corresponding pre-pandemic baseline periods used for comparison.

description	test period	baseline period
lockdown period	April 2020	15 September–15 October 2019
Omicron wave period	January 2022	January 2020

### 2.2. Data source

Mobility data gathered from mobile device GPS traces was provided for the purposes of this study by the data analytics company Pathzz (https://www.pathzz.com/). Pathzz is a consumer intelligence platform that uses mobility signals from opted-in smartphone devices in order to provide movement analytics, quantifying how people interact with spaces. The data are used by government organizations, private businesses, investment banks and research firms. For comparison with local population characteristics, users are assigned a statistical area as a home location (statistical area level 1 (SA1), Australian Statistical Geography Standard (ASGS) 2016 [40]). Home locations are assigned to devices based on where they are located most often between the hours of 18.00 to 8.00 Monday through to Friday and for the entire weekend period (Saturday and Sunday). By aggregating to home locations, mobility trends can be analysed with respect to aggregate statistical data from the Australian Census and other sources. In Australia, Pathzz data are derived from approximately 17 million unique devices per year, although the number of devices accounted for at a given time varies owing to fluctuating user numbers, use of multiple devices by individuals and device upgrades.

Using the Pathzz platform, a user begins by defining the destinations and time periods of interest. The Pathzz platform then provides the number of visits by home location, over all SA1 regions within 50 km of the specified destination. Visits are counted when a unique device enters the user-specified geospatial boundary during the specified period, and a maximum of one visit per calendar day is recorded for each device.

Compared to alternate sources of aggregate mobility data, Pathzz provides a flexible and high-resolution database. The capacity to define customized sets of destination locations allowed us to apply our analysis to entire urban regions, at high spatial resolution. Simultaneously, Pathzz protects the privacy of individual devices by restricting analysis to aggregate home locations. Pathzz does not provide individual device trajectories or link visits to personal information associated with devices (other than putative home regions) which helped us manage the risk of inadvertently violating user privacy during our study. Owing to the spatial heterogeneity of SES, and the high spatial resolution of the data, our study benefited from increased variance of SES between regions which would otherwise be lost owing to spatial aggregation.

### 2.3. Indices of economic resources, and education and occupation

The Australian Bureau of Statistics (ABS) publishes a set of four indices referred to as Socio-Economic Indexes for Areas (SEIFA) that quantifies the relative social and economic advantages and disadvantages of statistical regions at various spatial scales [37]. We use the 2016 edition of SEIFA. The indices are: Relative Socio-economic Advantage and Disadvantage (IRSAD), Index of Relative Socio-economic Disadvantage (IRSD), Index of Education and Occupation (IEO) and Index of Economic Resources (IER). Both IRSAD and IRSD reflect advantages and disadvantages in terms of combined social and economic factors. On the other hand, IEO and IER comprise variables that reflect only social advantage/disadvantage and economic advantage/disadvantage, respectively. ER and EO indices do not have any common component variables. Because ER and EO indices examine non-overlapping sets of variables, we chose them for our study of mobility trends by SES.

Specifically, the component variables of EO relate to educational qualifications and skill level of occupation, while those of ER are income, rental expenses and home ownership. A region will have a low EO score if: (i) many individuals are without educational qualifications, employed in low-skilled occupation, or unemployment is high; and (ii) few individuals are highly qualified or employed in highly skilled occupations. A region will have a low ER score if there are: (i) many households that have low income or pay low amounts of rent; and (ii) few households with high income or few people who own their home [37]. SEIFA indices are composed of variables that reflect the aggregate features of a spatial unit such as the percentage of home ownership in an area, or the percentage of individuals with a tertiary qualification. For the analysis and results presented in this study, we stratify the ER and EO scores for the SA1 regions of each city separately, into local decile bands. This is the method recommended by the ABS for using SEIFA indices [41].

SEIFA indices are composed of variables that describe the aggregate socio-economic characteristics of an area and not of all the individuals living in that area. For instance, one of the component variables of ER is ‘per cent of people aged 15 years and over who are unemployed’ and of EO is ‘per cent of people (in the labour force) unemployed’ [37]. There can be considerable variation in the SES characteristics of people living in an area [42]. The index scores are ordinal and the difference or ratio between the SEIFA scores of two areas does not have a straightforward interpretation [37].

### 2.4. Analysis of mobility data from Pathzz

**Figure 3.**
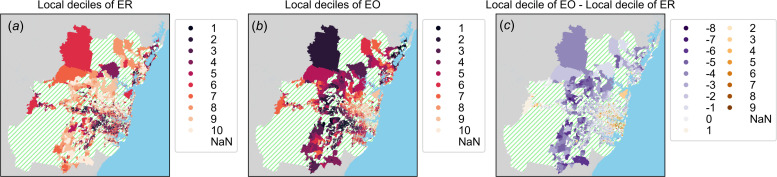
Choropleth maps of the Greater Sydney urban region with colours corresponding to in-sample decile scores for socioeconomic indices of ER (*a*), of EO (*b*), and (*c*) difference in decile scores of EO and ER. (*a*, *b*) Dark colours correspond to areas with lower SES and green-hatched regions were not included in the analysis because their coverage could not be computed. The grey colour shows land outside the greater urban region and blue coloured areas as water bodies. Note that while ER and EO are strongly correlated, areas with high EO and low ER tend to be located closer to the interior of the city, while regions with high ER and low EO tend to be located towards the urban fringe. This is also shown in (*c*) where we see a radial band of regions where ER and EO tend to be correlated (lighter colours). Similar spatial trends are observable in the Greater Melbourne area (see the electronic supplementary material, figure S4).

**Figure 4.**
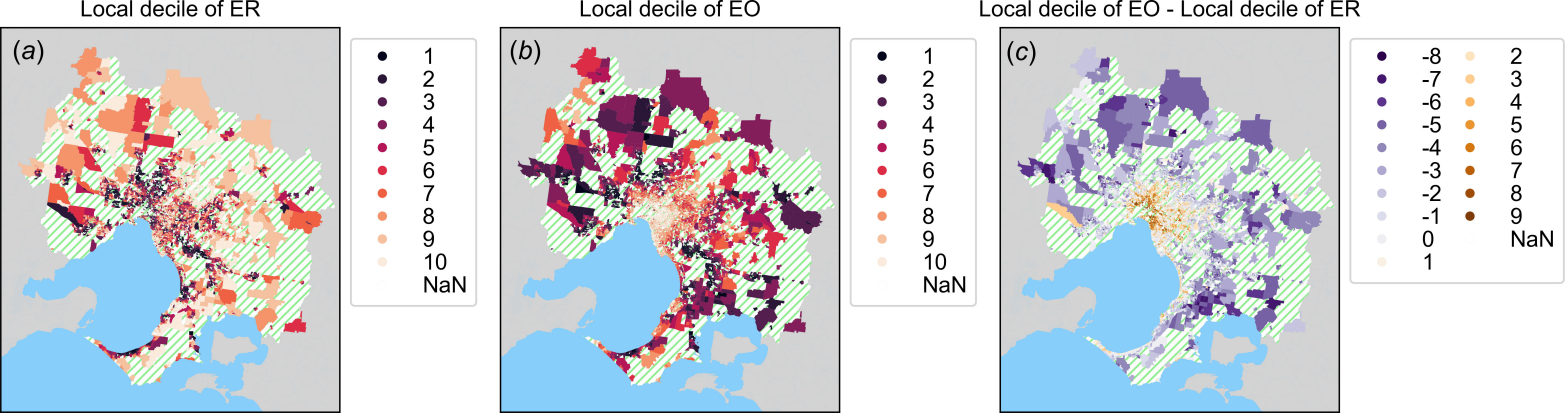
Choropleth maps of the Greater Melbourne urban region with colours corresponding to in-sample decile scores for socioeconomic indices of ER (*a*) of EO (*b)*, and (*c*) difference in decile scores of EO and ER. (*a*, *b*) Dark colours correspond to areas with lower SES and green-hatched regions were not included in the analysis. Green-hatched pattern (NaN in the legend) indicates those regions which were excluded from the analysis because their coverage could not be computed. Grey colour indicates land that is outside the Greater Melbourne region while the blue colour indicates water bodies. Note that while ER and EO are strongly correlated, areas with high EO and low ER tend to be located closer to the interior of the city, while regions with high ER and low EO tend to be located towards the urban fringe. This is also shown in (*c*) where we see a radial band of regions where ER and EO tend to be correlated (lighter colours).

For aggregation and processing of mobility data, space was divided into two different sets of partitions for origin locations (SA1 partitions) and destinations (destination zone (DZN) partitions, Australian Statistical Geography Standard). SA1 regions contain resident populations between 200 and 800 people, with a mean of approximately 400 (electronic supplementary material, figure S2). The spatial extent of SA1 and DZN regions depends on population density and is typically much greater on the urban fringe than in the central urban regions (figure 3 and figure 4). DZN regions spatially partition workplace locations and were originally designed for use in the analysis of place-of-work surveys included in the Australian Census. These partition schemes are described by the ABS ASGS [40].

For each DZN partition, data were collected from the Pathzz platform by specifying a time period (corresponding to the test periods and respective baseline periods) and the unique spatial polygon describing the DZN of interest. After specifying the DZN geometry, visitation counts from all SA1 regions were downloaded from Pathzz in the format of a ‘data analytics report’, which provides aggregate counts of all visits into the specified region that originated from each SA1 partition with a 50 km radius, over the specified time period. This count data from the entire set of DZN regions within Greater Sydney and Greater Melbourne was then disaggregated by origin to generate a full origin-destination matrix structured as a directed edge list (SA1 → DZN). Finally, these pairwise counts were reaggregated as mean counts per day by origin.

**Figure 5.**
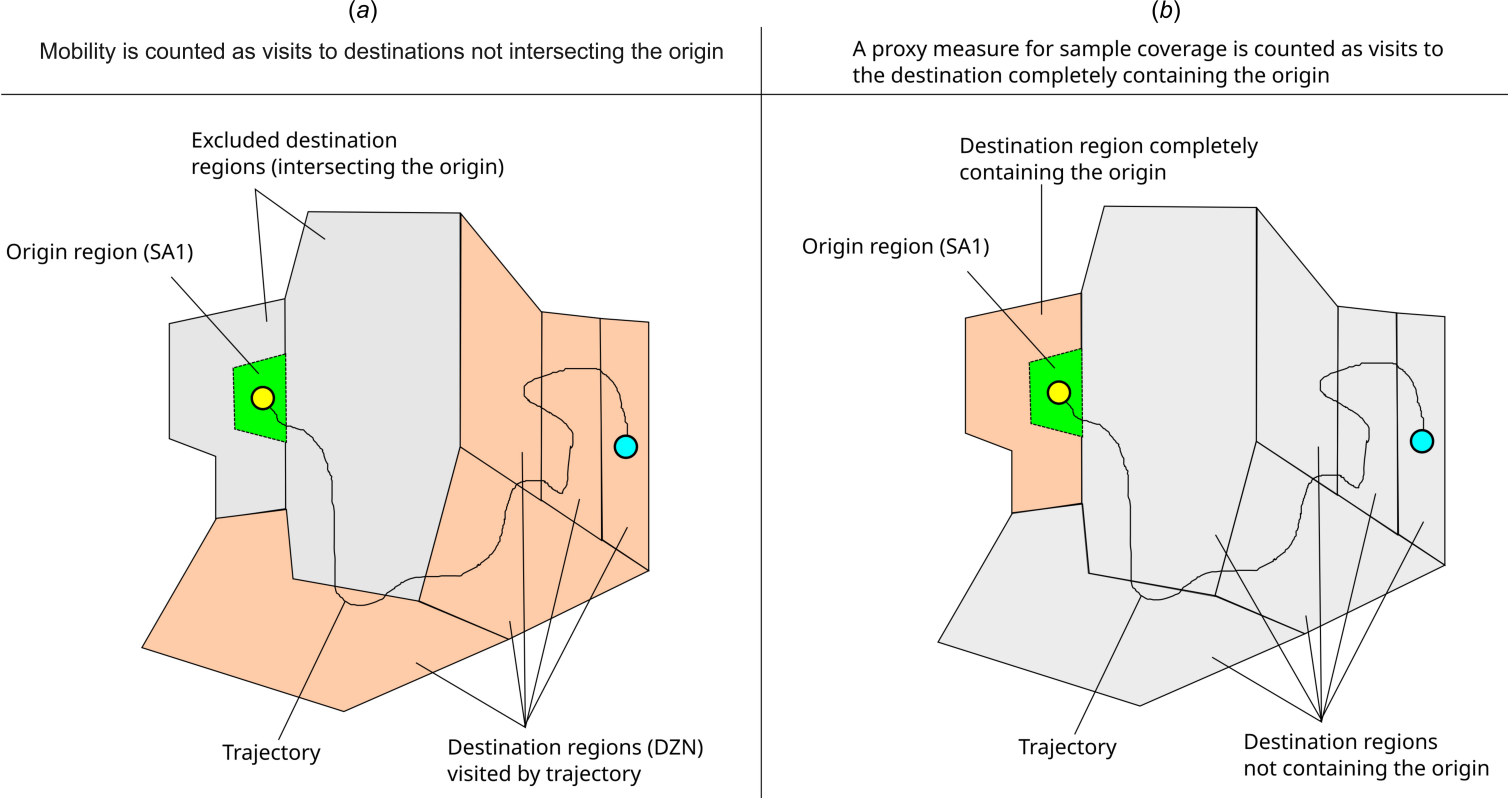
Schematic representations of mobility data aggregation for: (*a*) the method for computing counts of visits between origin regions and destination regions. A single individual trajectory is represented for illustrative purposes. Visits counted between the origin SA1 region and destination zones correspond to the number of trajectory points located within each DZN. The set of DZN regions intersecting the origin SA1 region are excluded from the count to avoid including spurious visits owing to GPS jitter and local movement within origin regions. (*b*) Sample coverage is computed as the number of trajectory visits counted within the destination area completely containing the origin region.

Within a DZN region, a visit of any duration is recorded and counted with equal weight. For this reason, adjacent and intersecting SA1 and DZN partition pairs were excluded from our analysis of mobility trends (figure 5*a*). Unlike other sources of mobility data (such as that provided by Meta’s data-for-good program), which are temporally filtered and can be interpreted as origin-destination matrices, the data described here should be interpreted as ‘pass-through’ counts (i.e. a visit should not be interpreted as a trip from an origin region and an individual’s final destination). If an individual passes through multiple DZN regions during a single day, they will be counted once in each region through which they pass. For this reason, we restrict our analysis to the comparison of visit counts, aggregated by origin SA1, relative to baseline (i.e. we implicitly assume that the rate at which origin-destination journeys are double-counted depends on the origin, but not on the time period over which data are aggregated).

For each origin region and time period of interest, we assess changes in mobility as the log-transformed ratio of trip counts during test and baseline periods, expressed as proportions of sample coverage over each time window:


(2.1) $$\lambda_{\text{mob}}(\text{SA1})=\ln\frac{A_{\text{SA1}}}{B_{\text{SA1}}}\;,$$

where


(2.2) $$\begin{array}{lcr} & A_{\text{SA1}}=\frac{\sum_{\text{DZN}}V(\text{S}\text{A}\text{1}\to \;\text{D}\text{Z}\text{N},T_{\text{test}})}{C(\text{SA1},T_{\text{test}})}\;, & \end{array}$$

is the number of visits V recorded for an SA1 origin region, summed over all non-intersecting destinations and averaged per day over the duration $\Delta t_{\text{test}}$ of the period of interest $T_{\text{test}}$ (either after the introduction of stay-at-home orders, or during re-opening), divided by the sample coverage C recorded over the same period and


(2.3) $$\begin{array}{lcr} & B_{\text{SA1}}=\frac{\sum_{\text{DZN}}V(\text{S}\text{A}\text{1}\to \;\text{D}\text{Z}\text{N},T_{\text{base}})}{C(\text{SA1},T_{\text{base}})}\;, & \end{array}$$

is the same measure for the corresponding pre-pandemic baseline interval $T_{\text{base}}$.

#### 2.4.1. External validation

To examine whether or not the data acquired from Pathzz was consistent with independent measures of mobility trends, we compared it to intersection pass-through data available through the Victorian Department of Transportation [43–45]. This external validation is described in full in the electronic supplementary material. Robust positive correlations were found for all periods, indicating that the mobility data acquired from Pathzz are generally consistent with other sources of mobility data.

## 3. Results

### 3.1. Overall mobility trends

**Figure 6.**
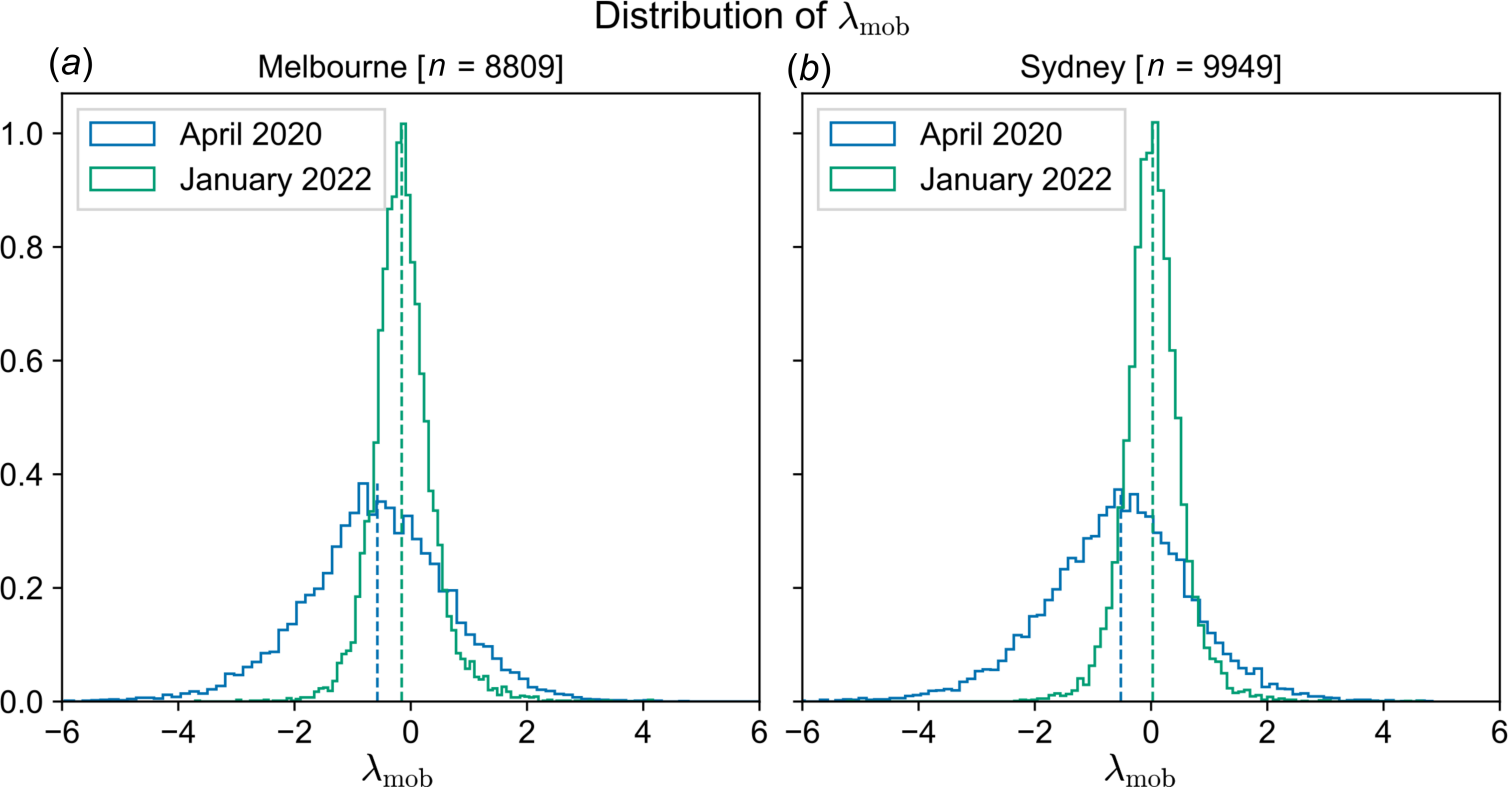
Histograms over SA1 regions of λ_mob_, the log-transformed ratio of mobility levels during COVID-19 periods and baseline periods. Blue traces correspond to the initial period of stay-at-home orders in April 2020, and green traces correspond to the Omicron surge and re-opening period in Jan 2022. The vertical dashed lines show the median value of the distribution with the corresponding colour.

Following the introduction of stay-at-home orders, median mobility decreased substantially in both urban regions, as shown by histograms of $\lambda_{\text{mob}}$ over SA1 partitions (figure 6). These distributions also show high variability in $\lambda_{\text{mob}}$, demonstrating that while mobility decreased overall, large numbers of SA1 regions exhibited higher mobility during lockdown, relative to baseline.

After the relaxation of mobility restrictions, international travel bans and regulations on social gatherings (the re-opening phase), there was a large wave of COVID-19 cases attributed to the Omicron variant. Overall changes in mobility are shown as distributions over SA1 partitions (figure 6), which shows a slight median decrease in Melbourne, and no significant change in median mobility in Sydney. Distributions for both regions demonstrate substantial variability, with approximately even proportions of SA1 regions showing higher or lower mobility relative to baseline.

### 3.2. Mobility as a function of socioeconomic indices

**Figure 7.**
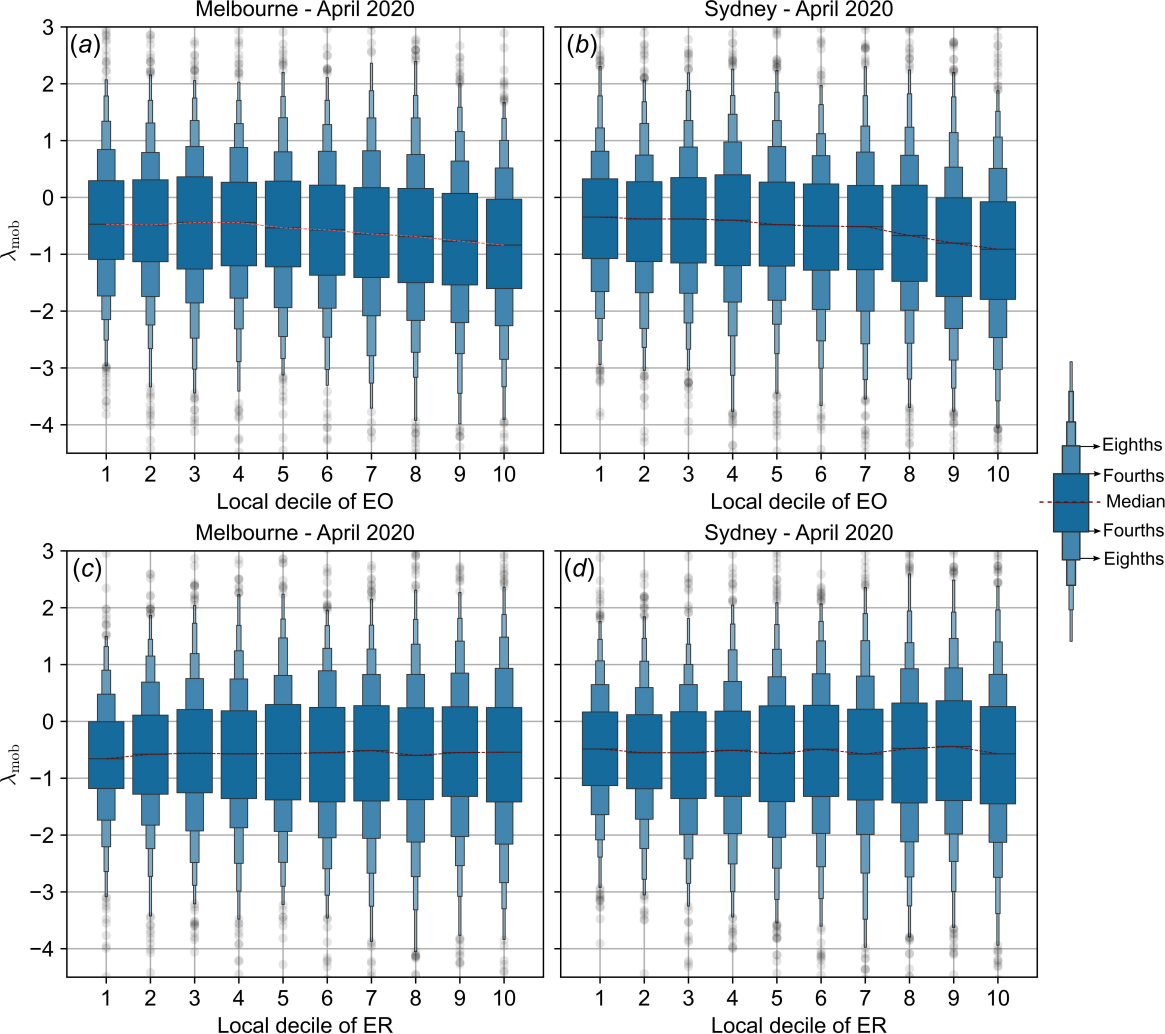
Mobility trends during stay-at-home orders as a function of socioeconomic indices. Summary statistics of λ_mob_, the log-transformed ratio of mobility relative to baseline, are shown as a function of EO index (*a,b*), and as a function of ER index (*c, d*). The letter-value plots show distribution summary statistics over SEIFA-stratified SA1 partitions of Australian cities of Melbourne (*a, c*) and Sydney (*b, d*) during April 2020. The central horizontal line within each box represents the median for the corresponding SEIFA decile, the two horizontal lines immediately above and below the median indicate the quartile bounds ('fourths'), the next set of boxes show the octile bounds ('eighths') and so on. See [46] for more details about letter-value plots.

**Figure 8.**
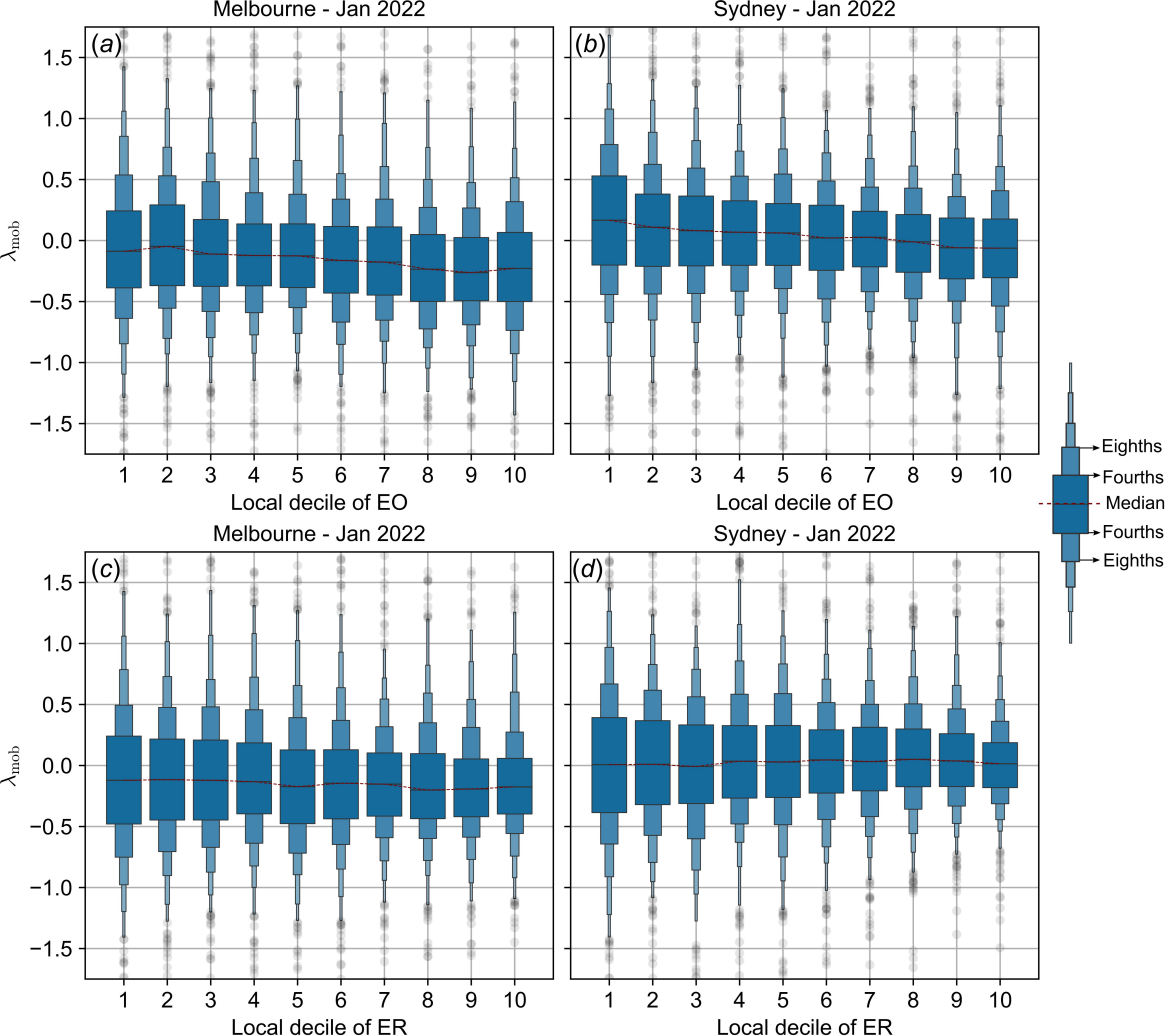
Mobility trends during the re-opening phase (and Omicron wave) as a function of socioeconomic indices. Summary statistics of λ_mob_, the log-transformed ratio of mobility relative to baseline, are shown as a function of EO index (*a,b*), and as a function of ER index (*c, d*). Letter-value plots show distribution summary statistics over SEIFA-stratified SA1 partitions of Australian cities of Melbourne (*a, c*) and Sydney (*b, d*) during January 2022. The central horizontal line within each box represents the median for the corresponding SEIFA decile, the two horizontal lines immediately above and below the median indicate the quartile bounds ('fourths'), the next set of boxes show the octile bounds ('eighths') and so on. See [46] for more details about letter-value plots.

To analyse the trends in mobility changes with socioeconomic indices, we stratify SA1 regions into local decile bands for EO and ER and plot decile medians (figures 7 and 8). During April 2020, in both Melbourne and Sydney, the median $\lambda_{\text{mob}}$ declines with increasing EO decile but does not vary with ER decile. We quantify these trends in decile medians by assessing their monotonicity with decile rank (Mann–Kendall (MK) test, table 2; [47,48]). This analysis demonstrates visible downward trends and significant monotonicity of median $\lambda_{\text{mob}}$ with EO decile rank (higher EO ratings correspond to lower median mobility relative to baseline, figure 7*a*,*b* and table 2).

**Table 2.** Monotonicity (Mann–Kendall) test results between SEIFA deciles and the median values of $\lambda_{\text{mob}}$. (For 10 data points, the Mann–Kendall score can take odd integer values between −45 (monotonically decreasing) and +45 (monotonically increasing).)

		greater Melbourne		greater Sydney	
period	SEIFA	Mann-Kendall score	*p-*value	Mann-Kendall score	*p-*value
April 2020	local decile of EO	−37	1.28 × 10^−2^	−45	8.30 × 10^−5^
	local decile of ER	25	3.18 × 10^–2^	−1	1.00
Jan 2022	local decile of EO	−39	6.77 × 10^−4^	−43	1.72 × 10^−4^
	local decile of ER	−33	4.21 × 10^−3^	19	1.07 × 10^−1^

In Melbourne, while there is no visible trend in $\lambda_{\text{mob}}$ as a function of ER decile, the monotonicity result reveals an increasing trend, significant at p=0.032 (table 2), but the magnitude of this trend is relatively smaller when compared to that of the EO decile. In Sydney, we find no trend in $\lambda_{\text{mob}}$ as a function of ER (with no visible trend and no monotonic relationship with decile medians).

During the re-opening phase in January 2022, while overall reductions in mobility are much less pronounced than during the initial phase of restrictions, the decline of $\lambda_{\text{mob}}$ with EO decile remains observable (figure 8*a*,*b*). Likewise, no clear trend is visible between $\lambda_{\text{mob}}$ and ER, for either urban region (figure 8*c*,*d*). However, the MK test of monotonicity between decile medians of $\lambda_{\text{mob}}$ and decile ranks reveals a slightly decreasing trend in Melbourne that is not observed in Sydney where a slight, but non-significant increase is observed (table 2). As in the case of April 2020, this decreasing trend is smaller in magnitude compared to the trend with EO deciles.

**Figure 9.**
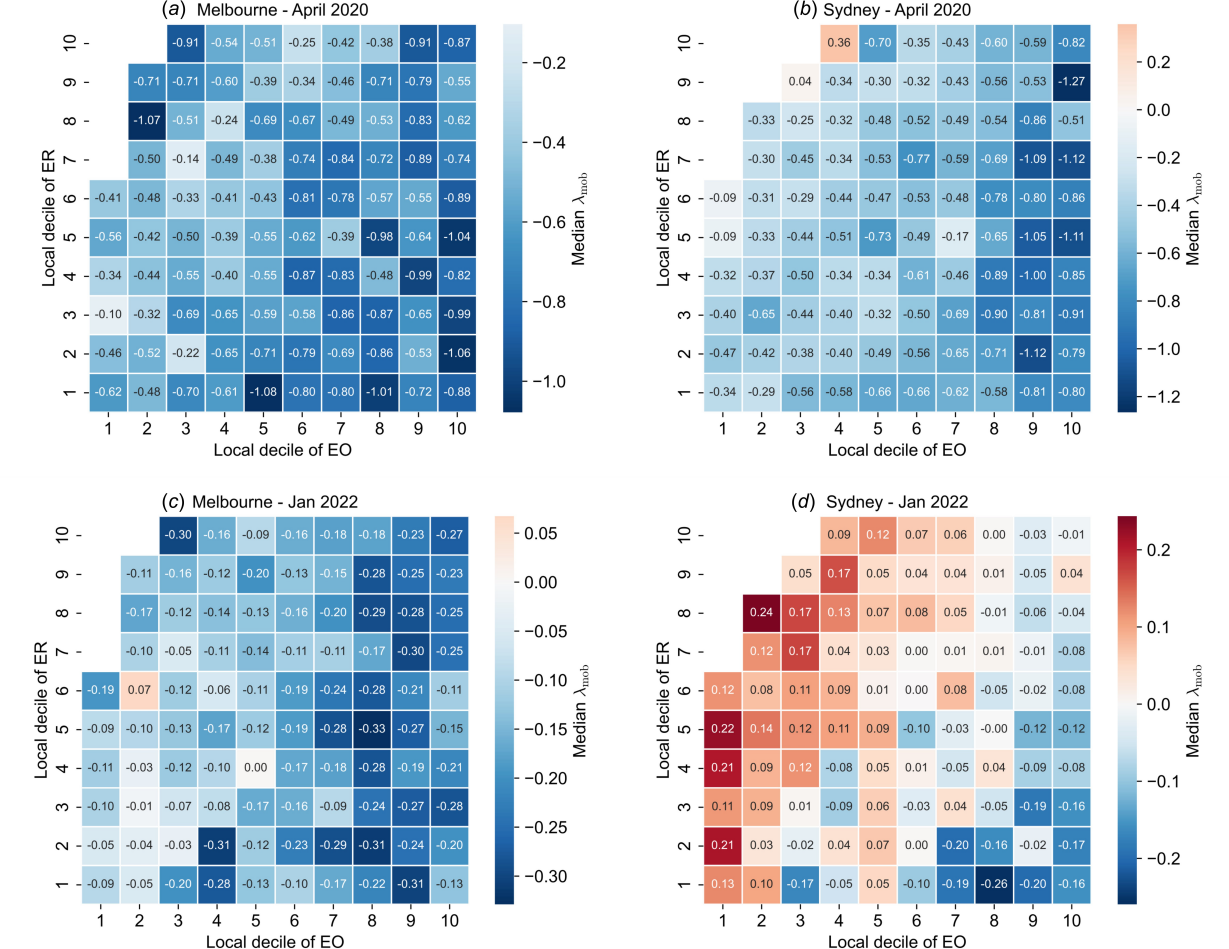
Heatmaps of median λ_mob_ (log-transformed ratio of mobility relative to baseline), jointly stratified by deciles of the IER and IEO. Mobility response during the initial period of stay-at-home orders is illustrated for Melbourne (*a*) and Sydney (*b*), and the re-opening phase in Melbourne (*c*) and Sydney (*d*). Red shades correspond to increased mobility relative to baseline, light shades correspond to near-baseline mobility and blue shades correspond to decreased mobility.

The general observation, visible in both periods, that trends in mobility behave qualitatively differently with ER and EO conflicts with the observation that ER and EO are themselves correlated (figure 2). To explain the observed deviation from the expected behaviour, we performed a joint analysis of mobility over both ER and EO strata. Joint trends of $\lambda_{\text{mob}}$ with ER and EO indices are shown as heatmaps in figure 9, which provides median $\lambda_{\text{mob}}$ values for SA1 regions jointly stratified by EO and ER decile. The joint trends of mobility with EO and ER differ between the two urban regions. In Melbourne, while $\lambda_{\text{mob}}$ tends to decrease with EO within ER deciles, there are no clear relationships between $\lambda_{\text{mob}}$ and ER within EO deciles for either period (figure 9*a*,*c*). For Sydney, both periods demonstrate jointly monotonic trends: $\lambda_{\text{mob}}$ decreases for higher EO within ER deciles and increases for higher ER within EO deciles (figure 9*b*,*d*). The joint trend is most apparent in figure 9*d*. The number of SA1 regions in each joint ER–EO category is shown in the electronic supplementary material, figure S3.

### 3.3. Statistical modelling

The primary results of this study are derived from qualitative analyses of the median trends with SES deciles as shown in figures 7, 8 and 9. To further quantify the qualitative trends shown above, we evaluated the relationship between SEIFA indices and mobility changes using three simple statistical models. Our objective in doing so was to test the statistical robustness of median trends and to determine if the trends in mobility with SEIFA indices are biased owing to spatial effects.

**Figure 10.**
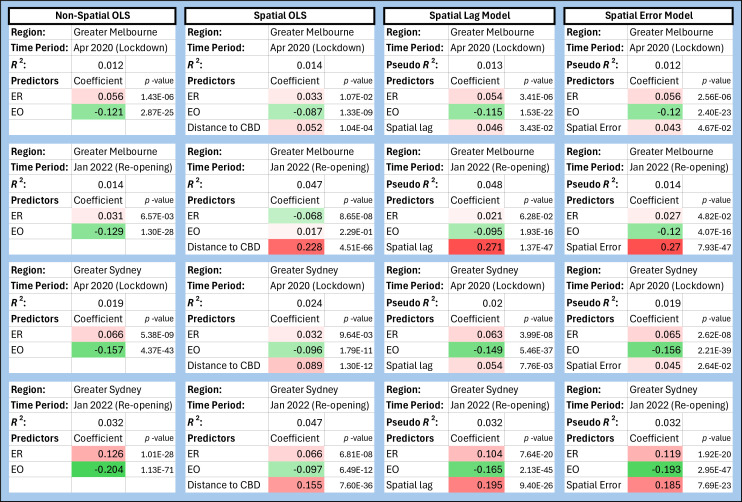
Summary of statistical models used. Results from four regression models with different predictor variables are shown: non-spatial ordinary least squares (OLS) with ER and EO, spatial OLS with ER, EO and distance of SA1 from the central business district (CBD), a spatial lag model and a spatial error model with ER and EO. This table shows the value, coefficients for each predictor variable and their *p*-values. The table entries for significant coefficients are highlighted according to the direction and magnitude of the trend, red for increasing and green for decreasing.

We start with a simple linear regression model where ER scores and EO scores are used as predictor variables for $\lambda_{\text{mob}}$. During both time periods and in both urban regions, $\lambda_{\text{mob}}$ shows a weakly decreasing trend with EO scores (consistent with our decile-stratified analysis) (Melbourne April 2020: β=−0.121, Melbourne January 2022: β=−0.129, Sydney April 2020: β=0.157, Sydney January 2022: −0.204, all p-values $<10^{-24}$), and a very small increasing trend with ER scores, which was not observed in our analysis of decile-stratified trends (Melbourne April 2020: β=−0.056, Melbourne January 2022: β=−0.031, Sydney April 2020: β=0.066, Sydney January 2022: −0.126, all p-values $10^{-3}$) (see figure 10, Non-Spatial OLS).

Next, we fitted three models that include spatial effects: (i) linear regression with distance of SA1 to the central business district (CBD) as an additional variable; (ii) a spatial lag model; and (iii) a spatial error model [49,50] (CBD refers to the city centre or downtown: the CBD areas of Melbourne and Sydney are characterized by high densities of businesses and residences). Our analysis of model (i) demonstrates that the distance to CBD is a significant predictor of $\lambda_{\text{mob}}$ for both urban regions and time periods. For the re-opening period (January 2022), it is a better predictor of $\lambda_{\text{mob}}$ than EO and is a similar or weaker predictor of $\lambda_{\text{mob}}$ during the lockdown period (April 2020). Despite this, EO and ER retain their trends and relative importance, except in the case of Melbourne during re-opening, where $\lambda_{\text{mob}}$ decreases with ER, and the trend with EO is non-significant after including distance to CBD (see figure 10, Spatial OLS). Scatter plots of the variables in this model show that EO is strongly anti-correlated with distance to CBD (see the electronic supplementary material, figures S7–10). In the spatial lag model, spatial lag is significant for both time periods and urban regions, however, we note that including spatial lag does not dramatically improve the model quality as quantified by Akaike information criterion (AIC) scores (electronic supplementary material, table S4). Spatial lag is a better predictor of $\lambda_{\text{mob}}$ than EO during re-opening, but the decreasing trend of $\lambda_{\text{mob}}$ with EO is retained. The spatial error model also preserves the broad trends in $\lambda_{\text{mob}}$ with ER and EO (see figure 10, Spatial Lag and Spatial Error models) and does not improve the AIC score substantially over the non-spatial OLS model (electronic supplementary material, table S4).

These models show that spatial effects (either distance to CBD, spatial lag or spatial error) can, in certain cases, account for variance in $\lambda_{\text{mob}}$ such that the weight of socioeconomic indicators in the regression is reduced. The scatter plots show a clear anti-correlation between EO and distance to CBD. While the statistical models weaken the results from the qualitative analysis of median trends to a varying degree, the key result (the counter-intuitive observation that ER and EO had differing effects on behaviour change) remains intact.

## 4. Discussion

We examined trends of mobility behaviour during two turning points of the COVID-19 pandemic in Australia as functions of two different indices of SES. During the initial period of stay-at-home orders during April 2020, we found that mobility decreased substantially overall and declined as a function of the index of EO, but found no relationship between mobility and the index of ER. Surprisingly, while overall reductions in mobility were much less substantial during the re-opening phase (and Omicron wave) in January 2022, mobility trends as a function of socioeconomic indicators were similar, decreasing for higher indices of EO, but stable as a function of ER. In Sydney, we found a prominent joint dependence of mobility on both socioeconomic indicators, producing a scenario in which regions with high ER, but low EO exhibited the highest mobility levels relative to pre-pandemic baseline.

To interpret these findings, we consider them from the frame of reference provided by the COM-B model of behaviour, which relates capability (C), opportunity (O) and motivation (M), to individual behaviour (B) [51]. Capability refers to attributes of individuals that make behaviours possible in principle. These could include having sufficient mental capacity (e.g. understanding and memory) and physical capacity (e.g. physical hardware or physiological ability) to carry out the behaviour. Opportunity refers to environmental factors that facilitate the behaviour, such as proximity to locations where the behaviour can be performed, or access to the financial resources required to undertake actions necessary for the behaviour. Motivation refers to the desire to change behaviour, which can be influenced by observations and information. Motivation is mediated by opportunity and capability in the sense that if a behaviour is perceived as difficult because the conditions facilitating it are not present, the desire to do it may decrease. Finally, behaviour refers to the activities of individuals, and behaviour change occurs when an individual switches from one set of activities to another. The COM-B model emphasizes the effects of feedback between behaviours enacted, and the COM factors affecting the tendency to change behaviour.

With respect to the COM-B model, the socioeconomic indices we analysed can be considered components of capability because they describe characteristics of individuals that mediate behaviour but are stable over long timescales. Situational assessment can provide insight into the opportunity and motivation components. Opportunity to change behaviour can be facilitated through support policies such as the economic support packages provided by the Australian Government during the initial period of stay-at-home orders, and we credit these with the notable lack of correlation between ER and mobility changes. We observed that all income strata demonstrated similarly large decreases in mobility during this crucial period, in contrast to findings from other contexts [17–19].

On the other hand, it appears the capability factors associated with education level and occupation status were associated with decreased mobility, which was observed for both locations during the initial restrictions and Omicron surge. This result is rather unique—while other studies have observed correlations between drops in mobility and education level, these past studies investigated scenarios in which mobility restrictions and COVID-19 transmission were simultaneously present [20,21]. By contrast, our study examines a period during which mobility restrictions were absent, and the drivers of mobility behaviour change are more likely attributable to discretionary responses to epidemic prevalence and the perception of risk. This result may reflect that people with higher education levels were more capable of staying informed about the risks associated with infection, helping to motivate risk avoidance behaviour. The role of occupational status is probably related to the higher proportion of people with high-skilled occupations who are able to work from home while maintaining secure employment conditions [52]. In addition, exemptions to stay-at-home restrictions were given to maintain the workforce in essential industries, many of which employ low-skilled occupations and are associated with lower EO indices (with some exceptions, such as healthcare workers) [53].

There are several limitations to our analysis that must be taken into consideration and warrant future work to validate these findings with the analysis of additional data. Namely, because the data we analysed were not curated specifically for the purpose of this investigation, five methodological limitations arose that could not be fully accounted for:

- the trajectory data underlying our analysis was not subject to temporal filtering and aggregation;
- sample coverage could only be computed for a subset of regions;
- home locations in mobility data may not correspond to true residence location;
- bias in the user base with SES; and
- assumptions about behavioural changes.

First, because the trajectory samples were not temporally filtered, the counts of visits we analysed do not represent easily interpreted quantifiers of origin-destination travel behaviour. In our analysis, individuals are counted multiple times as they traverse the urban landscape (once per day, for each DZN they visit), and the interpretation of our observations rests on the assumption that the rate of double-counting did not change between the baseline periods and test periods analysed in our study. Second, because the calculation of sample coverage required an SA1 to be completely contained by a single DZN, it could only be computed for a subset of regions. Many of the excluded regions were located in the urban fringe where SA1 and DZN geometries tend to be more extensive, with complex boundaries. Further work should account for sample coverage from all regions so that exclusions can be minimized. Third, because a data-driven approach was used to classify the residential locations associated with de-identified device trajectories, there is no guarantee that the home location is a true representation of the user’s residential address. For this reason, the association between the SES of residential partitions (SA1 regions) and home locations of tracked devices is subject to unknown bias. Fourth, although the *Pathzz* platform draws data from a large number of devices, there is no guarantee that it represents an unbiased sample from the spatial partitions. Since, the data are not linked to the users’ SES, we could not adjust for possible biases. Fifth, in addition to these limitations arising from the nature of the data, the interpretation of the log-transformed ratio of pass-through counts ($\lambda_{\text{mob}}$) requires the assumption that individuals changed their behaviour only by changing the frequency at which they travel while the trajectories of their trips remained consistent. This limitation has implications for the interpretation of spatial effects identified in our statistical models, and addressing it should form the basis of future work (see below).

The statistical modelling presented in §2.3 revealed further limitations of our qualitative analyses of the joint trends with socioeconomic variables. We found that spatial effects were significant in some cases, reducing the explanatory power of SEIFA indices on mobility changes. Specifically, distance of a region from the CBD zone was shown to be a better predictor during re-opening (January 2022) in both urban regions, than their SEIFA scores. This indicates that urban spatial effects can confound analysis based on socioeconomic indicators.

As mentioned in the discussion of limitations above, the importance of this finding for our conclusions rests on the validity of the key assumption that changes in mobility were dominated by changes in travel frequency, with negligible changes to the spatial patterns of travel. If this assumption is valid, then we can interpret the influence of spatial variables as common cause effects: socioeconomic constraints drive the spatial distribution of populations, as well as the sensitivity of those populations to pandemic-related behavioural stimulus. However, if the spatial patterns of travel were dramatically altered by pandemic-related stimulus, the value of $\lambda_{\text{mob}}$ calculated from our analysis would contain a bias produced by assuming the number of pass-through counts per trip is the same for baseline and test periods.

While resolving this question is beyond the scope of the present study, its importance for the interpretation of results such as ours motivates future work examining the spatial and temporal features of mobility patterns during periods of social change.

## 5. Conclusion

Future implementation of equitable public health responses to pandemics such as COVID-19 requires a better understanding of how socioeconomic factors influence behaviour. In this study, we found that mobility decreased with indicators of education and occupation (the EO index) for small spatial partitions of the urban landscape in the cities of Melbourne and Sydney, Australia. This correlation was observed both during stay-at-home orders, when case incidence was low, and during the re-opening phase, when case incidence was highest and restrictions were not in place. In contrast to observations in other national contexts, wealth (as quantified by the index of ER) was not found to be correlated with mobility trends for either period. As the indices for ER and EO are generally correlated, these contrasting trends indicate opposing effects by each index. We tentatively conclude that these observations reflect three underlying causes: (i) the availability of economic support to facilitate compliance with stay-at-home orders; (ii) the spatial clustering of essential workers who remained mobile during the initial period of restrictions; and (iii) the capacity of those with highly skilled employment to work from home. Additionally, we speculate that while the combined effects of low economic resources and high education level led to risk-averse behaviour during the re-opening phase, the opposite was true for those with high economic resources and low education level. These findings can help facilitate the design of equitable and effective pandemic interventions in future scenarios by identifying factors which can be used to help predict the heterogeneous behavioural responses to policy implementation and relaxation.

## Data Availability

All data and code necessary for reproducing our main analysis are provided in the linked repository [55]. This includes aggregate mobility counts and coverage levels for all SA1 regions for all time periods analysed. We have not released raw data downloaded from the Pathzz platform. Supplementary material is available online at [56].
